# Effectiveness of *Lonomia* antivenom in recovery from the coagulopathy induced by *Lonomia orientoandensis* and *Lonomia casanarensis* caterpillars in rats

**DOI:** 10.1371/journal.pntd.0006721

**Published:** 2018-08-16

**Authors:** Ida S. Sano-Martins, Camila González, Isabelle Valle Anjos, Juana Díaz, Luis Roberto C. Gonçalves

**Affiliations:** 1 Laboratório de Fisiopatologia, Instituto Butantan, São Paulo–SP, Brazil; 2 Centro de Investigaciones en Microbiología y Parasitología Tropical (CIMPAT), Facultad de Ciencias, Universidad de los Andes, Bogotá, Colombia; Institut de Recherche pour le Développement, BENIN

## Abstract

In South America, accidental contact with Lepidoptera larvae can produce a diversity of reactions that vary from dermatological problems to severe hemorrhagic syndromes, such as those caused by contact with caterpillars of the genus *Lonomia* (Saturniidae). *Lonomia* venom can alter the hemostatic system and lead to renal failure, internal and brain bleeding, and in severe cases, death. The only specific treatment available for these envenomations is the *Lonomia* Antivenom (LAV) produced by the Butantan Institute, in Brazil, using an extract of *Lonomia obliqua* scoli as the antigen. LAV has been used to treat exposure to other *Lonomia* species across South America. However, no experimental studies have been performed to test the efficacy of LAV in neutralizing the venom of species other than *L*. *obliqua* found in Southern Brazil. In this study, we tested the effectiveness of LAV in reversing the hemostatic disturbances induced by injecting *Lonomia casanarensis* (Lca) and *Lonomia orientoandensis* (Lor) scolus extracts into rats and compared the effects to the case of *L*. *obliqua* (Lob) scolus extract-induced envenomation. Lca and Lor caterpillars were collected in Colombia, and some of them were reared to adults for identification. The Minimum Defibrinating Doses (MDD) of Lca and Lor were estimated. Rats were injected (i.d.) with a dose of 3 MDD per rat of each scolus extract and treated (i.v.) with 1.5 mL of LAV or 1.5 mL of saline. Twenty-four hours after the treatment, the fibrinogen levels and platelet counts had recovered to the hemostatic levels in the groups treated with LAV. The groups treated with the saline solution had fibrinogen levels and platelet counts at non-hemostatic levels. Thromboelastometric analyses confirmed these results. In conclusion, the results showed that LAV is effective at neutralizing the envenomation induced by Lca and Lor spine extracts in rats and restoring hemostasis.

## Author summary

A specific antivenom therapy is recommended for treating envenomation caused by venomous animals, such as snakes, spiders, scorpions, and *Lonomia* caterpillars. Studies with snake bites have shown that antivenoms are effective for treating the envenomation caused by snakes of the same genus, even when the antigen composition used to generate the antivenom did not include the specific venom. The *Lonomia* antivenom produced by the Butantan Institute to treat envenomations caused by contact with *Lonomia* caterpillars only uses the extract of *Lonomia obliqua* scoli as the antigen. However, there are reports of other species of *Lonomia* in South America that demonstrate biological activities that are different from those observed in response to *L*. *obliqua* venom, but which cause similar symptoms in human envenomation. No information is available regarding the neutralizing capacity of the *Lonomia* antivenom in these other species. This study showed that the *Lonomia* antivenom efficiently reversed the hemostatic disorders induced in rats by injection of extracts of the scoli of *L*. *casanarensis* and *L*. *orientoandensis* from Colombia. These results support the possibility of using this antivenom for treating envenomations caused by other *Lonomia* species in humans.

## Introduction

Several families within the Lepidoptera (moths and butterflies) produce harmful venoms. One group, the family Saturniidae, includes a genus, *Lonomia* (Saturniidae: Hemileucinae), of particular public health concern [1–3].

As a part of their defense mechanisms, *Lonomia* caterpillars are gregarious and form groups of over fifty individuals, have cryptic colors, and feed at night on host plant leaves [4]. The spines of *Lonomia obliqua* Walker, 1855 caterpillars are associated with a gland that is a single large cell in the subapical region [5]. Venom is released when the spine breaks after touching or penetrating a rigid surface, such as the human skin, allowing venom absorption [4–6].

Symptoms of the envenomation that results from contacting *Lonomia* caterpillars can vary from intense local pain or a burning sensation at the site of contact, to renal failure and intracerebral and intraperitoneal hemorrhages that can lead to death [7–10]. Several studies of the composition of the scolus extract and toxicity have been conducted in Brazil and Venezuela. Regarding their epidemiological importance, two species are involved in accidents: *Lonomia achelous* Cramer, 1777 and *L*. *obliqua* [9,11–13].

*Lonomia obliqua* is distributed in the southern region of South America, including Brazil, Uruguay, Paraguay, and northern Argentina, while *L*. *achelous* is present in the northern part of South America in Brazil, Ecuador, Peru, Venezuela, Guyana, and Colombia [14].

The first records of envenomation by *Lonomia* caterpillars in South America came from Venezuela in 1967 and involved *L*. *achelous* caterpillars [8,15,16]. Brazil and Venezuela have reported high numbers of cases of contact with *L*. *obliqua* and *L*. *achelous* [8,9], and in recent years, Argentina [17–19] and Peru [20] have also indicated an increasing number of accidents and fatalities.

In Colombia, most cases are recorded in the Casanare Department, but there is no available information regarding the real number of accidents in Colombia or the species that were involved. The official records noted 32 cases between 2000 and 2010, with two deaths [4,21,22].

Since 1994, the recommended treatment of envenomation from contacting *Lonomia* caterpillars in Brazil is the use of *Lonomia* antivenom (LAV), which is produced using an extract of the *L*. *obliqua* scoli as the antigen [23]. In Venezuela, the treatment is based on the use of antifibrinolytics, such as aprotinin, or the transfusion of fibrinogen or cryoprecipitate [24]. In Colombia, treatment LAV has been successful, although the efficacy of this antivenom for Colombian *Lonomia* species and treatment protocols have not been yet established [21,22].

In this context, this study aimed to test the effectiveness of the *Lonomia* antivenom produced in Brazil by the Butantan Institute for treating rats envenomed by an injection of scolus extracts from two caterpillar species collected in Colombia, *Lonomia casanarensis* Brechliin, 2017 and *Lonomia orientoandensis* Brechliin, 2011.

## Materials and methods

### Ethics statements and animals

All procedures involving the use of animals were performed under the resolutions of the National Council for the Control of Animal Experimentation (Conselho Nacional de Controle de Experimentação Animal—CONCEA) and approved by the Ethics Committee of Animal Use the Butantan Institute (protocol 1353/14 and 63000505/17). Male Wistar rats that weighed 250–300 g were supplied by the Central Animal Laboratory of the Instituto Butantan. Rats were housed in plastic cages, within a room in which the temperature was controlled to between 22 and 23°C. Rats were exposed to a 12:12 h light:dark cycle and were allowed free access to food and water.

### Scolus extracts and *Lonomia* antivenom

**Fig 1:**
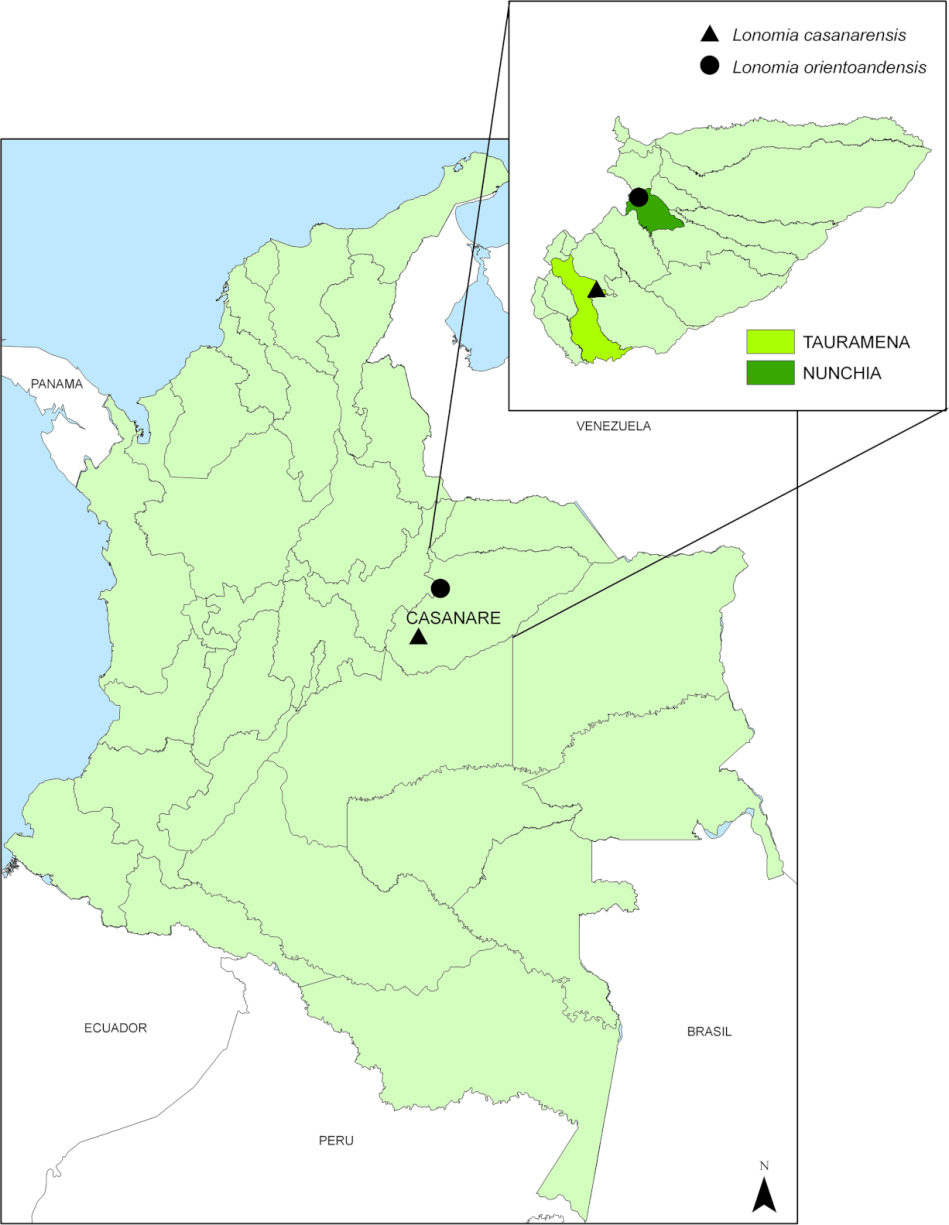
Geographic location of caterpillar’s collection sites. Map of Colombia highlighting the Casanare department and showing the localities where *Lonomia casanarensis* (triangle) and *Lonomia orientoandensis* (circle) caterpillars were collected. This figure was built using ArcGis 10.5 software, licensed to Universidad de Los Andes; the layers are available for free: elevation from the Hydro 1K available at https://www.usgs.gov/ and political division by the National administrative department of statistics (Dane by its acronym in Spanish) at https://geoportal.dane.gov.co. This figure doesn't include any satellite image from a copyrighted source.

Colonies of *L*. *orientoandensis* (Lor) and *L*. *casanarensis* (Lca) in the 5^th^ and 6^th^ instars were collected in the Department of Casanare, Colombia. Lca caterpillars were collected in the municipality of Tauramena (4.90773°S, -72.5437°W) and Lor caterpillars in the municipality of Nunchía (5.64629°S, -72.2011°W) (Fig 1). The colonies were transported to Bogotá in plastic boxes supplied with host plant leaves. The extraction of the venom was carried out in the Centro de Microbiología y Parasitología Tropical (CIMPAT) at the Universidad de los Andes. The extraction of the scoli was performed following the methods of Dias da Silva *et al*. (1996) [11] with some modifications. The *Lonomia* caterpillars were anesthetized using low temperature. Once anesthetized, their bristles were cut off at the insertion of the tegument and maintained at 4ºC until preparing the extract. The scoli were ground with a mortar and mixed with cold phosphate-buffered saline (PBS), pH 7.4, to achieve a 10% (w/v–mg/mL) final extract solution. The solution was centrifuged at 3000 *g* for 20 min at 4ºC. The supernatant of the extract was stored in 1.0 and 0.5 mL aliquots at -80ºC. Frozen aliquots were transported on dry ice to the Butantan Institute (São Paulo, Brazil) in polystyrene boxes and kept at -80°C until testing.

The Lob scoli extracts and *Lonomia* antivenom (LAV, batch:1304060) were provided by Butantan Institute (São Paulo, Brazil). One vial of LAV, which contains ten mL of LAV, neutralizes 3.5 mg of the *Lonomia obliqua* extract, according to the producer and the Brazilian pharmacopeia. The potency of the antivenom takes into account the neutralization of the coagulant action of the *Lonomia obliqua* scolus extract in mice [25].

### Species identification of *Lonomia* caterpillars

Some of the collected caterpillars were reared to adults for morphological identification. The identification was performed based on the descriptions of the species [26–28] and the revisions of the genus *Lonomia* by Lemaire [14]. DNA barcode confirmation was carried out using caterpillars that had been preserved in 70% alcohol. The DNA was extracted from one leg of the specimens following the standard protocol described by Ivanova *et al*. (2006) [29]. The pair of primers LepF1 and LepR1 was used to amplify a 658 bp fragment of the COI gene [30]. The PCR products were then sequenced using the Sanger method, and the reads were edited and then aligned using Geneious software [31]. The obtained sequences were compared to all the *Lonomia* sequences (~800) in the Barcode of Life Data Systems (BOLD; www.boldsystems.org).

### Experimental protocol

Minimum Defibrinating Dose (MDD). The MDD is the minimum protein concentration that induces blood incoagulability at least one hour after i.d. injection of scoli extract in rats. It was determined according to the method described by Dias da Silva *et al*. (1996) [11], with some modifications. Male Wistar rats (250–300 g) were separated into five groups, and each group (n = 3) was injected (i.d.) with a specific concentration (12.5, 25, 50, 100 or 150 μg/rat) of extract from one of the three species. Depending on the timing of the onset of symptoms, one or two hours after the envenomation, rats were anesthetized with isoflurane (Cristalia, S. Paulo), and blood was collected by puncture of the abdominal aorta for the determination of the whole blood clotting time [32].

Envenomation and neutralization assay. Seven experimental groups were used to test the efficacy of LAV treatment. The challenge doses corresponded to 3MDD of scoli extracts of each species.

The following groups were used:

1) The control group was not envenomed and was treated with sterile saline 0.9% (1.5 mL, i.v.).
2) Group envenomed i.d. with Lob (150 µg/rat) and treated with LAV (1.5 mL, i.v.) one hour later.
3) Group envenomed i.d. with Lob (150 µg/rat) and treated with sterile saline 0.9% (1.5 mL, i.v.) one hour later.
4) Group envenomed i.d. with Lor (450 µg/rat) and treated with LAV (1.5 mL, i.v.) two hours later.
5) Group envenomed i.d. with Lor (450 µg/rat) and treated with sterile saline 0.9% (1.5 mL, i.v.) two hours later.
6) Group envenomed i.d. with Lca (300 µg/rat) and treated with LAV (1.5 mL, i.v.) two hours later.
7) Group envenomed i.d. with Lca (300 µg/rat) and treated with sterile saline 0.9% (1.5 mL, i.v.) two hours later.

Blood sampling and coagulation analysis. Twenty-four hours after treatment, rats were anesthetized with isoflurane (Cristalia, S. Paulo), and blood was collected from abdominal aorta. The blood was then transferred at a ratio of 9:1 (v:v) to tubes that contained 13 mM sodium citrate and 1% of EDTA with 2% of LAV to neutralize the venom activity in the sample. The platelet counts were determined in EDTA-anticoagulated blood using an automated cell counter (Mindray BC-2800 Vet, Nanshan, Shenzhen, China). The fibrinogen level was assayed in citrated plasma stored at -80°C using colorimetric methods [33]. Whole blood was collected to test the general aspect of coagulation through thromboelastrometric analyses using a Rotation Trombelastogram analyzer ROTEM coagulation system and software (Pentapharm GmbH, Germany). The Trombelastogram analyzer ROTEM gives a graphical representation of clot formation and subsequent lysis when it occurs. The measurements were performed in the 4 channels for one hour. The following parameters were determined: NATEM (plasma recalcification time), EXTEM (extrinsic pathway), INTEM (intrinsic pathway), and FIBTEM (fibrinogen ability to form the fibrin clot, without the influence of platelets).

Classical thromboelastometric parameters, such as the coagulation time (CT), clot formation time (CFT), maximum clot firmness (MCF) and alpha angle (α-angle), were analyzed for each assay.

### Statistical analysis

One-way ANOVA followed by the Tukey test was used to evaluate significant differences between the treated and non-treated groups. The data are expressed as the means ± SEM, and differences were considered statistically significant when the *P* <0.05. For these analyses we have used the software GraphPad Prism, version 7.04.

## Results

The morphological and barcoding parameters confirmed the classification of caterpillars collected in Colombia as *L*. *casanarensis* and *L*. *orientoandensis*.

The Minimum Defibrinating Doses of the scolus extracts of the Lca and Lor obtained after i.d. injection were 100 and 150 μg/ rat, respectively. The animals presented symptoms such as prostration, dyspnea and hemoglobinuria, and unclottable blood 2 h after the injection of extract. In rats injected with Lob, the same signs and changes in coagulation were observed 1 h after the envenomation, and the MDD for the scolus extract of Lob was 50 μg/ rat.

The challenge doses used for the LAV treatment experiments corresponded to 3MDD, which were 150 µg of Lob, 450 µg of Lor or 300 µg of Lca scolus extract per rat. The antivenom treatments were performed at the time of full onset of the signs and symptoms of envenomation, i.e., 1 h after Lob envenomation, and 2 h after Lca and Lor envenomation. The hemostatic parameters were tested 24 h after LAV treatment.

**Fig 2:**
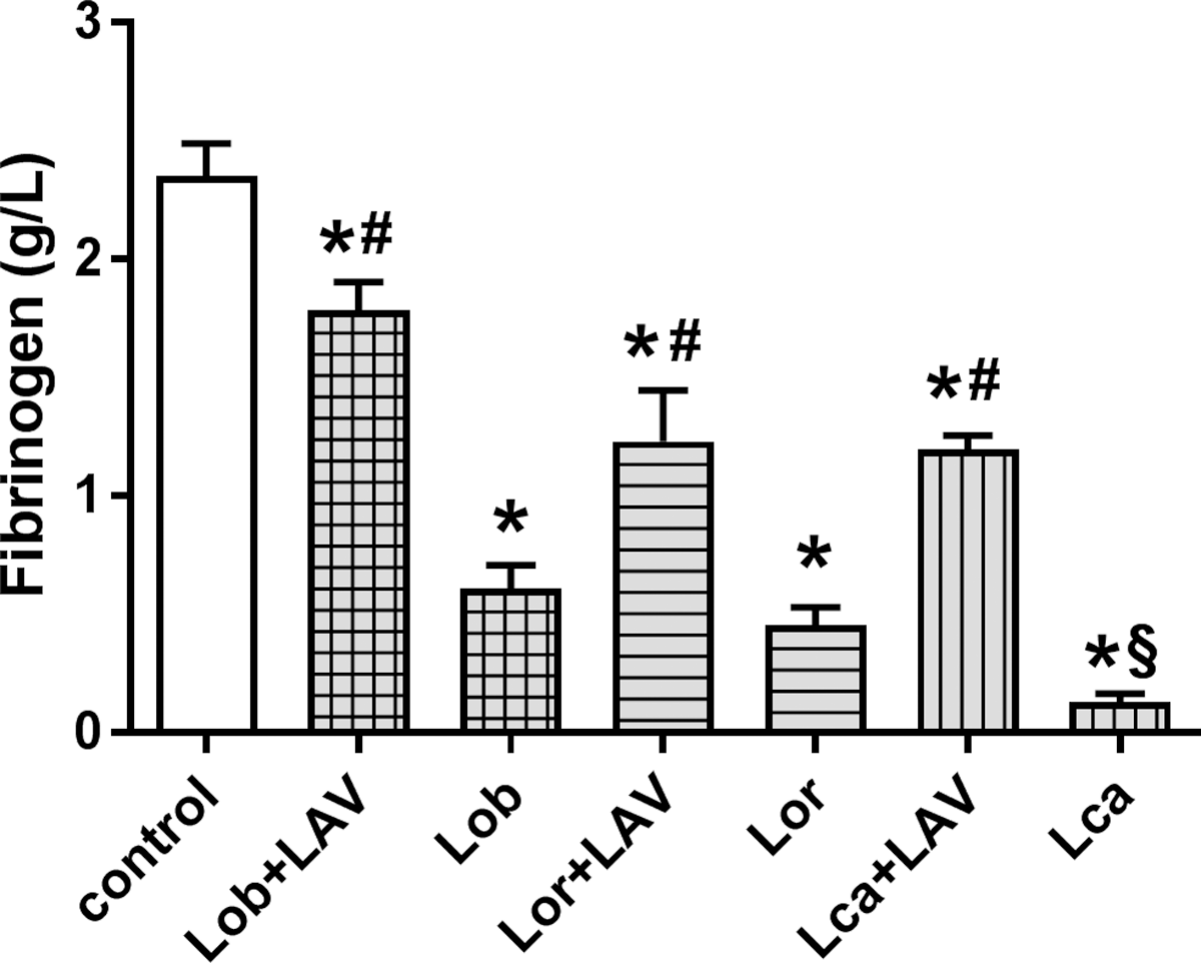
Recovery of fibrinogen levels in rats envenomed with *Lonomia* scoli extracts and treated with LAV, in comparison to envenomed but non-treated animals. The animals were injected i.d. with Lob (150 µg), Lor (450 µg), or Lca (300 µg), which corresponded to 3 MDD of each extract. One hour (Lob), or two hours (Lor and Lca) later, the animals were treated with the LAV or sterile saline (1.5 mL, i.v.), and blood was collected 24 h after the treatment. Fibrinogen was measured in the citrated plasma as described in the Material and Methods section. The results are expressed as the means±SEM, n = 6 rats/group. Significant differences are indicated as follows (p<0.05): (*) different from the control non-envenomed group; (#) different from the respective envenomed and saline-treated groups, and (§) different from the other groups.

Twenty-four hours after LAV treatment, none of the treated animals showed any signs or symptoms of the envenomation. The envenomed groups demonstrated a significant consumption of fibrinogen, whereas in the LAV-treated groups, the fibrinogen was at hemostatic levels, although it remained at levels below that of the control group. In the animals treated with LAV, the groups injected with Lor or Lca demonstrated fibrinogen levels lower than those observed in the Lob-injected group. Among the untreated groups, the animals injected with Lca demonstrated lower fibrinogen levels than any other animal group (Fig 2).

**Fig 3:**
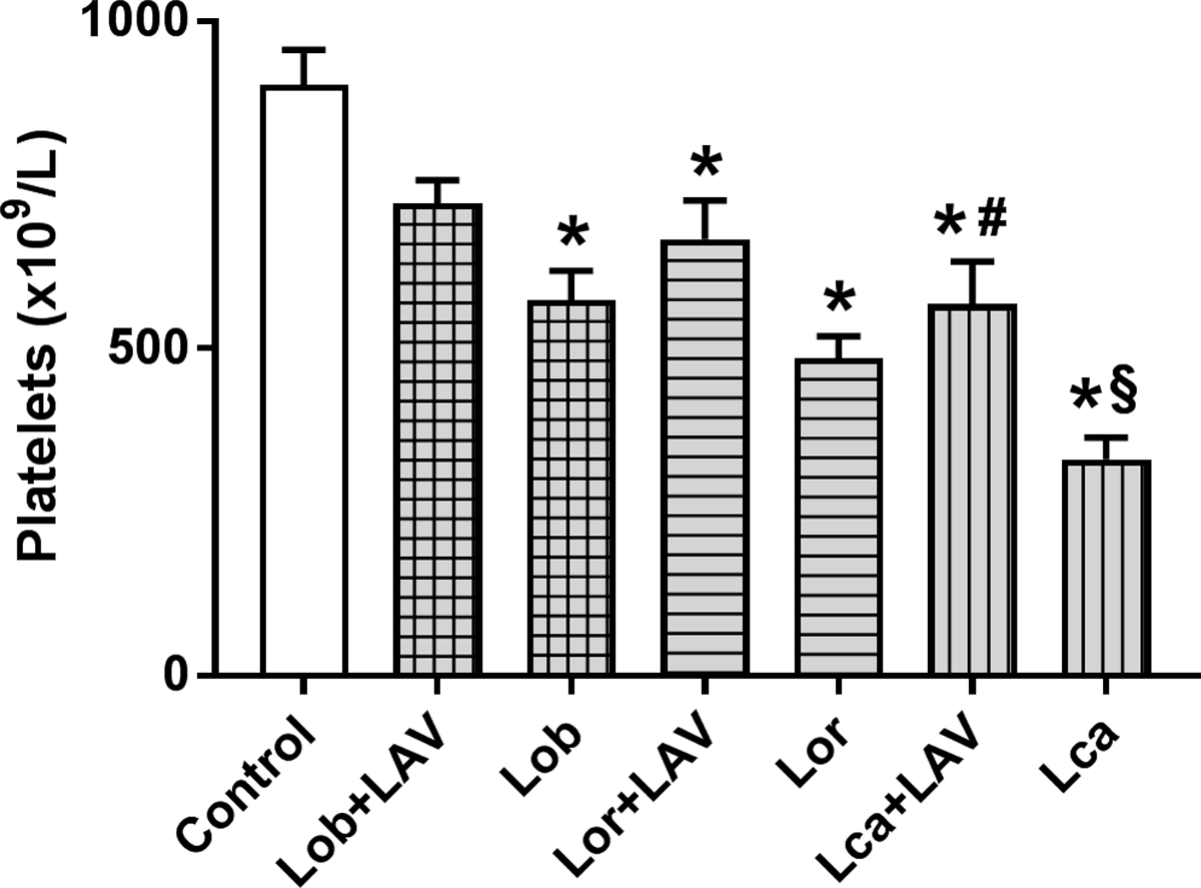
Recovery of platelet counts in rats envenomed with the *Lonomia* scoli extracts and treated with LAV, in comparison to envenomed but non-treated animals. Animals were injected i.d. with 3 MDD of each extract: Lob (150 µg), Lor (450 µg), or Lca (300 µg). One hour (Lob), or two hours (Lor and Lca) later, animals were treated with the LAV or sterile saline (1.5 mL, i.v.), and blood was collected 24 h after the treatment. The platelets were counted in EDTA-anticoagulated blood using an automated cell counter as described in the Material and Methods section. The results are expressed as the means±SEM, n = 6 rats/group. Significant differences are indicated as (p<0.05): (*) different from the control non-envenomed group; (#) different from the respective envenomed and saline-treated groups; and (§) different from the other groups.

Regarding the platelet counts, all of the envenomed animals demonstrated thrombocytopenia compared to the control groups. In the animals injected with Lca, the thrombocytopenia was more severe than that observed in the other groups. All of the groups treated with LAV showed a partial reversal of this effect (Fig 3).

**Fig 4:**
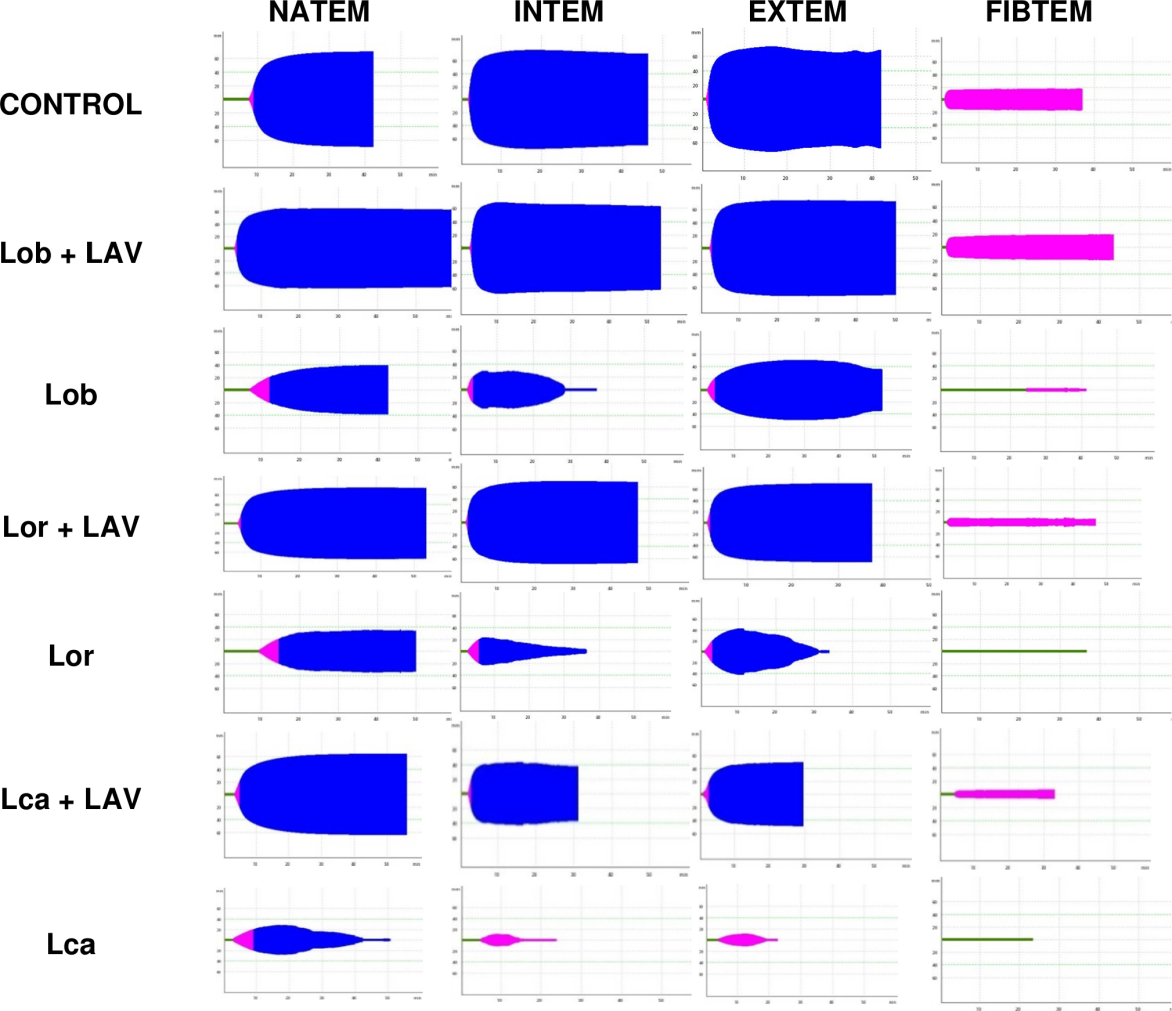
Thromboelastometry of rat blood samples collected 24 hours after the i.v. treatment with LAV or saline in rats envenomed (i.d.) with scoli extracts of *L*. *obliqua* (150 µg/rat), *L*. *orientoandensis* (450 µg/rat), *L*. *casanarensis* (300 µg/rat) caterpillars. Graphical representations of the assay in citrated whole blood with addition of 0.2 M calcium chloride and saline (NATEM) and specific reagents for the analysis of extrinsic pathway (EXTEM), intrinsic pathway (INTEM), or fibrinogen (FIBTEM). The control was a non-envenomed rat that was treated with *Lonomia* antivenom. The data were captured by the ROTEM software in the time interval of 40–60 min. Each set of graphs represents one animal in each experimental group.

The thromboelastographic profiles revealed that hemostatic disturbances were present in all of the analyzed parameters in the groups injected with the various scoli extracts. We chose the thromboelastographic profile of one rat as representative of each parameter of different groups. Fig 4 shows that in the envenomed groups that were treated with LAV, the thromboelastographic patterns were similar to those observed in the control non-envenomed animals. The profiles of these groups confirm the efficacy of the LAV treatment in restoring hemostasis.

**Fig 5:**
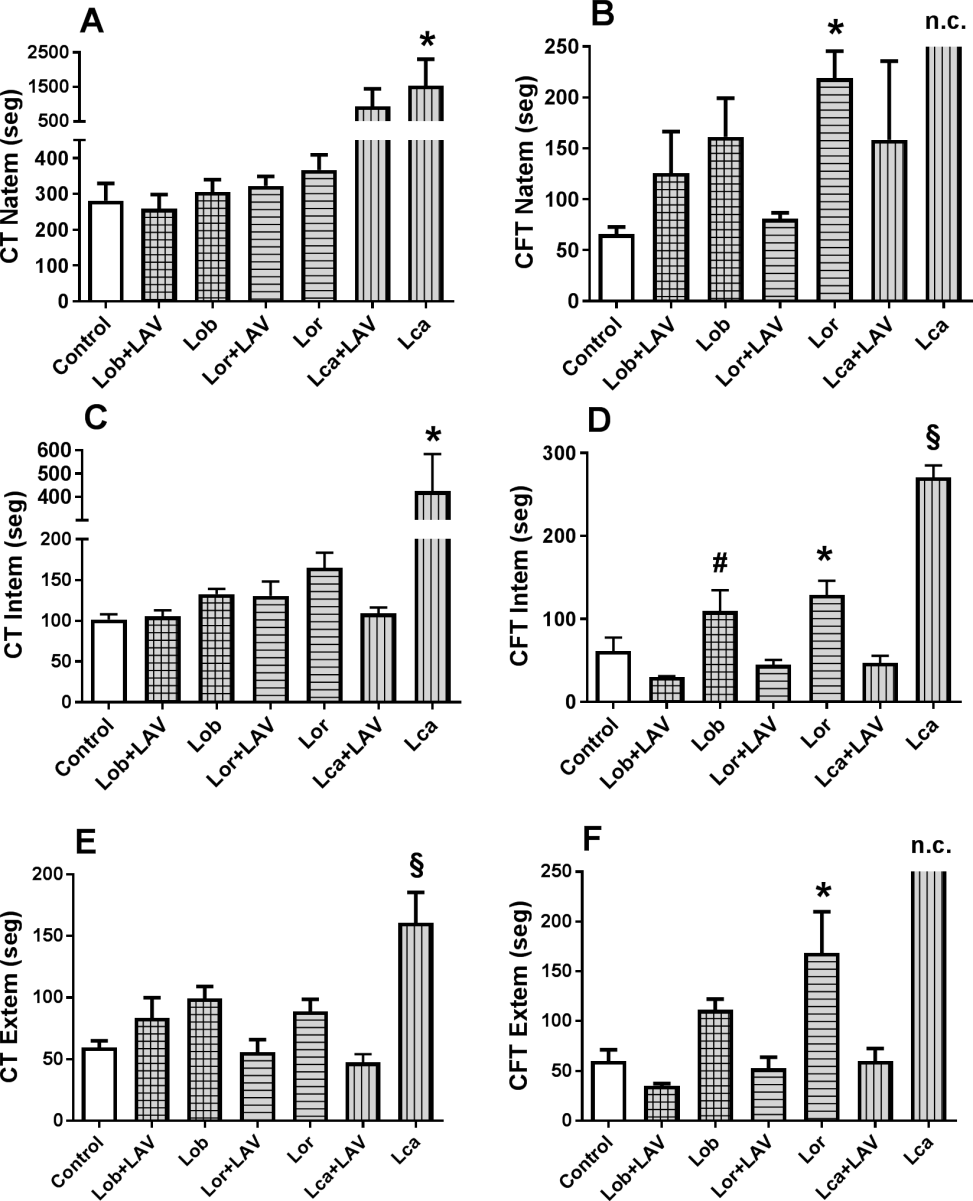
Quantitative values of the Clotting Time—CT (A, C, E) and Clot Formation Time–CFT (B, D, F) obtained from the thromboelastometry of NATEM (A, B), INTEM (C, D), and EXTEM (E, F) of rats envenomed with the *Lonomia* scoli extracts and treated with LAV, in comparison to envenomed but non-treated animals. The animals were injected i.d. with scoli extract of *L*. *obliqua* (Lob-150 µg), *L*. *orientandensis* (Lor-450 µg), or *L*. *casanarensis* (Lca–300 µg), which corresponded to 3 MDD (Minimum Defibrinating Dose) of each extract. One hour (Lob), or two hours (Lor and Lca) later, the animals were treated with LAV (1.5 mL, i.v.) or sterile saline, and blood was collected 24 h after the treatment. Thromboelastography was performed on citrated whole blood with the addition of 0.2 M calcium chloride (NATEM) or specific reagents for the analysis of the extrinsic pathway (EXTEM), or intrinsic pathway (INTEM). The control group consisted of non-envenomed rats that were treated with the *Lonomia* antivenom. The data were captured by the ROTEM software in the time interval of 40–60 min. n = 6 rats/ group. Significant differences are indicated as follows (p<0.05): (*) = different from the non-envenomed control group and different from the respective group treated with the antivenom; (#) = different from the respective group treated with the antivenom, but not different from the non-envenomed control group; (§) = different from other groups; and (n.c.) = no clot.

In the analysis of NATEM, INTEM, and EXTEM, the quantitative values of CT did not differ among the groups, except for the Lca–envenomed group, which demonstrated the longest time (Fig 5A, 5C and 5E). This severe Lca envenomation was also observed in the CFT, but for this parameter, the LAV–treated groups showed a significant recovery when compared to the envenomed animals that were not treated. (Fig 5B, 5D and 5F).

**Fig 6:**
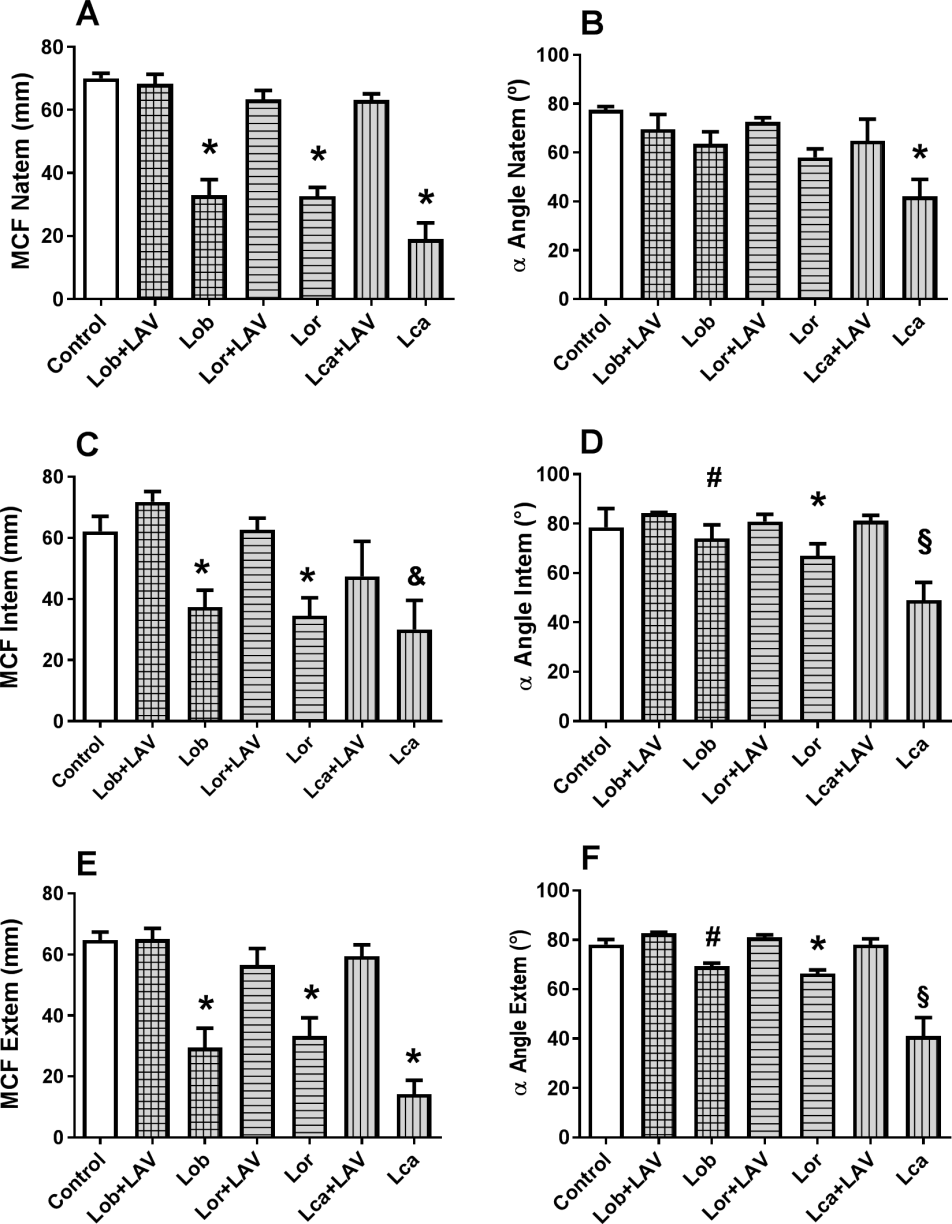
Quantitative values of the Maximum Clot Firmness—MCF (A, C, E) and Alpha (α) Angle (B, D, F) determined from the thromboelastometry of NATEM (A, B), INTEM (C, D), and EXTEM (E, F) of rats envenomed with the *Lonomia* scoli extracts and treated with LAV, in comparison to envenomed but non-treated animals. The animals were injected i.d. with scoli extract of *L*. *obliqua* (Lob-150 µg), *L*. *orientoandensis* (Lor-450 µg), or *L*. *casanarensis* (Lca–300 µg), which corresponded to 3 MDD (Minimum Defibrinating Doses) of each extract. One hour (Lob), or two hours (Lor and Lca) later, the animals were treated with the LAV or sterile saline (1.5 mL, i.v.), and blood was collected 24 h after the treatment. Thromboelastography was performed in citrated whole blood with the addition of 0.2 M calcium chloride (NATEM) or specific reagents for the analysis of the extrinsic pathway (EXTEM), or intrinsic pathway (INTEM). The control group consisted of non-envenomed rats treated with LAV. The data were captured by the ROTEM software in the time interval of 40–60 min, n = 6 rats/ group. Significant differences are indicated as follows (p<0.05): (*) = different from the non-envenomed control group and different from the respective group treated with the antivenom; (#) = different from the respective group treated with antivenom but not different from the non-envenomed control group; (§) = different from other groups; (&) = different from the non-envenomed control group, but not different from the respective antivenom-treated group; and (n.c.) = no clot.

Among the thromboelastographic parameters, the MCF values showed the most marked difference between the envenomed groups and the respective envenomed groups treated with LAV, which indicated the reversal of the changes caused by the envenomation (Fig 6A, 6C and 6E). The α-angle was normalized in the LAV-treated groups, and the Lca-injected group presented values lower than in all other groups (Fig 6B, 6D and 6F).

The results for FIBTEM (Table 1) showed significant changes in the values of CT, CFT, MCF and the alpha angle in the groups treated with LAV compared to the untreated groups. These parameters also indicate a more severe envenomation by Lca, but the treatment with LAV shows a tendency for these parameters to recover, as observed in the other animals envenomed by Lob and Lor.

**Table 1:** Coagulation parameters of the FIBTEM assay from thromboelastometry analysis of blood samples of different groups of rats. The animals (n = 6 rats/group) were injected i.d. with Lob (150 µg), Lor (450 µg), or Lca (300 µg), which corresponded to 3 MDD (Minimum Defibrinating Dose) of each extract. One hour (Lob), or two hours (Lor and Lca) later, the animals were treated with LAV or sterile saline (1.5 mL, iv), and blood was collected 24 h after the treatment. Thromboelastography was performed in citrated whole blood with the addition of 0.2 M calcium chloride and specific reagents for the analysis of the FIBTEM. The control group consisted of non-envenomed rats treated with LAV. The data were captured by the ROTEM software in the time interval of 40–60 min. n = 6 rats/ group.

FIBTEM	CT (s)	CFT (s)	MCF (mm)	α Angle (°)
**Control**	68.6 ± 58.6	278.2 ± 536.7	26.2 ± 20.9	77.0 ± 7.2
**Lob + LAV**	103.2 ± 97.3	0.0 ± 0.0	14.8 ± 2.9	61.6 ± 34.6
**Lob**	1537.2 ± 1507.2	0.0 ± 0.0	2.0 ± 1.8	0.0 ± 0.0
**Lor + LAV**	43.1 ± 34.2	114.3 ± 272.2	18.0 ± 27.0	42.5 ± 52.2
**Lor**	1240.0 ± 996.9	2.6 ± 6.5	7.1 ± 17.5	25.1 ± 61.4
**Lca + LAV**	520.6 ± 593.1	0.0 ± 0.0	6.2 ± 4.6	0.0 ± 0.0
**Lca**	2461.6 ± 832.5	0.0 ± 0.0	0.0 ± 0.0	0.0 ± 0.0

CT = clotting time, in seconds; CFT = Clotting firmness time, in seconds; MCF = Maximum clot firmness, in millimeters; Alpha Angle, in degrees.

## Discussion

For the first time, LAV efficacy was challenged against different *Lonomia* species, contributing from the Public Health perspective. In the present study, we used rats as an experimental model to examine the efficacy of the LAV in treating animals envenomed with extracts of the scolus from *L*. *orientoandensis* and *L*. *casanarensis* caterpillars from Colombia and compared these effects with the envenomation caused by *L*. *obliqua* caterpillars. We showed that LAV produced from *L*. *obliqua* scolus extract reversed the envenomation caused by the venom extracted from other *Lonomia* species, which supports the efficacy of this treatment in cases of human envenomation.

Diagnosis of envenomation by contact with *Lonomia* caterpillars is based on clinical signs and symptoms, and the hemorrhagic syndrome is based on consumption coagulopathy. In recent years, Argentina [17,18], Peru [20,34], and French Guiana [35] have reported accidents resulting from contact with *Lonomia* caterpillars, including fatalities. In Colombia, from 2005 to 2010, after an epidemiological surveillance protocol is established, the use of LAV was started, and since then, 32 cases have been reported, including two deaths[21]. In 2014, the Ministry of Health of Colombia declared a National Emergency due to the need for a treatment against envenoming with *Lonomia* caterpillars (resolution 01302 of 2014). Recently, a 40-year-old woman was envenomed by contact with a caterpillar, probably *Lonomia*, in French Guiana. She presented pain, systemic hemorrhage, coagulopathy with undetectable plasma fibrinogen, a high level of D-dimer, macroscopic hematuria, but normal platelets count and renal function. She received LAV in the eleventh days after the skin contact and she recovered 5 days after antivenom therapy, corroborating the efficacy of the *Lonomia* antivenom [35].

Here, we also have shown that *L*. *casanarensis* and *L*. *orientoandensis* might cause human envenomation since these species induced hemostatic disturbances in rats. The specimens were collected in Casanare, Colombia, a region where most recorded accidents, some fatal, have occurred. In the first known fatal accident in the region, the victim collected the caterpillars and after being reared to adults the species was identified as *L*. *achelous* based on the morphology of a female specimen [21]. This species is also known to be responsible for accidents in Venezuela [7]; however, after many tries to collect *L*. *achelous* in Colombia, this species still has not been found. Barcode analyses have allowed the species identification with similar morphological traits, so the diversity of *Lonomia* species involved in accidents has been underestimated. From this perspective, the identification of species involved in accidents is recommended [21,22].

In terms of venom composition, *L*. *obliqua* and *L*. *achelous* are the most well-known species within the genus that have toxic biological activities. Both species show coagulant activities including factor II and X activators [7,9] and kallikrein-kinin system activation [36]. *L*. *achelous* has a direct fibrinolytic activity [37] and inactivates blood clotting factors XIII [38] and V [39]. In *L*. *obliqua*, the described toxins have direct and indirect hemolytic activities [40], cardiotoxic, myotoxic and genotoxic activities [41], as well as effects on platelet adhesion and aggregation [42]. Some studies have shown that no fibrinolytic activity is present in *L*. *obliqua*, and the fibrinolytic activities are secondary to a blood coagulation disorder. Others have suggested the presence of fibrino(geno)lytic activities and identified transcripts of fibrinolytic toxins in *L*. *obliqua* [9,43–45].

Regarding the envenomation in rats, our results showed differences among species. The MDD varied among Lor and Lca, and incoagulability occurred two hours after the envenomation, while this effect appears one hour after Lob envenomation in rats. This difference could be due to the stronger procoagulant activity of Lob or the presence of hyaluronidase, which facilitates the passage of the venom through the dermis [46]. Further studies on the composition and biological activities of Lca and Lor extracts are needed to clarify this point. Characterization of the biological activities of Lor and Lca is our next goal, which is in progress.

For a better evaluation of the treatment with LAV, we compared its effects in a group of rats envenomed with *L*. *obliqua* scoli extract, whose antivenom is efficient in restoring hemostatic disorders in rats [47] and in injured patients [41,48,49]. We standardized the treatment based on the time of blood incoagulability induced by the scoli extract in each of the caterpillar species, and the challenge dose used was 3 MDD. The *L*. *obliqua* caterpillar scoli extract caused blood incoagulability at 1 h after envenomation with a lower dose (50 µg) than the Colombian caterpillar species. Thus, the challenge doses used were different among caterpillar species. However, the levels of fibrinogen in rats envenomed with Lob or Lor for 24 hours and treated with saline were similar, showing the same severity of envenomation. The level of fibrinogen in the Lca group was lower than the other two groups. Despite this, in all groups treated with LAV, the levels of fibrinogen and platelets recovered. However, recovery was faster for rats envenomed with Lob and Lor, who had hemostatic changes less intense than the Lca-envenomed group. Thus, our results show that LAV can neutralize the toxins *of L*. *orientoandensis* and *L*. *casanarensis* caterpillars from Colombia, although LAV is produced using only *L*. *obliqua* extract as antigen. Despite Lor and Lca is less active than Lob does not mean that the accidental envenomation by skin contact with these species should be less severe since fatal accidents have been registered in Colombia [22].

In our study, thromboelastometry showed that the hemostatic parameters normalized in the groups that were envenomed by the various *Lonomia* species and treated with LAV. Thus, analyses performed 24 h after treatment the NATEM, EXTEM, and INTEM results showed that the CT differed from the control group only in the group envenomed with Lca. The CFT (time at which the clot reached maximum viscosity) showed a particular variation according to the activation of coagulation by extrinsic (EXTEM) or intrinsic (INTEM) pathways. This parameter showed that there was sufficient fibrinogen to clot and to form a stable fibrin. The MCF describes the firmness of the clot. For this parameter, all the LAV–treated animals were equal to the control non–envenomed group while the saline-treated groups differed from those treated with LAV and the controls. Concerning the alpha angle, which shows the speed of the clot formation, the only group that differed from the control group was envenomed with Lca.

The FIBTEM, CT, CFT, alpha angle and MCF values of the saline-treated groups remained altered. It was not possible to define and analyze the CFT parameter by the software because the samples did not clot. MCF, the primary parameter for the evaluation of fibrinogen by the FIBTEM assay, was altered in the envenomed group. We observed that 24 hours after the LAV treatment, the CT values decreased to levels close to those saw for the control group. An increase of the alpha angle was obtained in the LAV-treated group. MCF showed values closer to that saw for the control, but still below the value considered normal. The CFT of the LAV-treated groups remained prolonged, showing that despite the LAV treatment, the clot that formed still had deficiencies in firmness and quality.

These results are consistent with the effectiveness of antivenoms against snake bites of the same genus, including species whose venom was not included in the antigen pool [50–52]. Various species of snakes belonging to the *Bothrops* genus show a high similarity in the composition of their venoms. The differences in the biological activities observed among several species of *Bothrops* are due to the quantitative variation of the proteins, and not to the lack of a protein class.

In conclusion, our results show that LAV can reverse the hemostatic disturbances caused by skin contact with other *Lonomia* species, including *L*. *orientoandensis* and *L*. *casanarensis* from the region of Casanare (Colombia). These results corroborate the successful treatment with LAV of a few accidents that occurred in Colombia, Argentina, Peru, and French Guiana, even when the antivenom was applied late. A double-blind clinical and laboratory study should be carried out in regions where similar accidents occur to test the need for production of an antivenom using antigens from other species of caterpillars.
